# Short- and medium-term outcomes of intracorporeal versus extracorporeal anastomosis in laparoscopic right colectomy: a propensity score-matched study

**DOI:** 10.1186/s12957-020-02112-2

**Published:** 2021-01-04

**Authors:** Chun-Kai Liao, Yih-Jong Chern, Yueh-Chen Lin, Yu-Jen Hsu, Jy-Ming Chiang, Wen-Sy Tsai, Pao-Shiu Hsieh, Hsin-Yuan Hung, Chien-Yuh Yeh, Jeng-Fu You

**Affiliations:** 1 Colorectal Section, Department of Surgery, Chang Gung Memorial Hospital, No. 5, Fuxing St., Guishan Dist, Taoyuan, 33305 Taiwan; 2 School of Medicine, Chang Gung University, No. 259, Wenhua 1st Road, Guishan Dist, Taoyuan, 33302 Taiwan

**Keywords:** Right hemicolectomy, Laparoscopic surgery, Propensity score, Resection margin, Intracorporeal anastomosis, Peritoneal recurrence

## Abstract

**Backgrounds:**

Though better short-term outcomes were frequently reported, differences in specimen parameters and the rate of subsequent peritoneal recurrence between intracorporeal anastomosis (IA) and extracorporeal anastomoses (EA) for laparoscopic right hemicolectomy have not been analyzed. We aimed to compare the pathologic differences and oncological outcomes between these two approaches.

**Methods:**

We retrospectively analyzed 217 consecutive patients who underwent laparoscopic right hemicolectomies from September 2016 to April 2018 and classified them into IA and EA groups, based on the approach used. Propensity score matching analysis was performed, after which 101 patients were included in each group with the patients matched for demographics, tumor stage, and localization.

**Results:**

The IA group had a longer operative time, shorter length of stay, shorter time to first flatus and tolerating a soft diet, and better pain scale scores at postoperative day 3. No inter-group differences in conversion, postoperative complication, mortality, or readmission rates were found. The IA group had a longer resected colon length (23.67 vs. 19.75 cm, *p* = 0.010) and nearest resected margin (7.51 vs. 5.40 cm, *p* = 0.010) for cancer near the hepatic flexure. There are comparable 3-year overall survival (87.7% vs. 89.6%, *p* = 0.604) and disease-free survival (75.0% vs. 75.7%, *p* = 0.842) between the IA and EA groups. The rate of peritoneal recurrence was similar between the two groups (5.9% vs. 7.9%, *p* = 0.580).

**Conclusions:**

The overall survival, disease-free survival, and the rate of peritoneal recurrence were comparable between the IA and EA procedures. IA ensures better recovery and comparable complications to EA and achieved a more precise tumor excision; thus, IA can be considered a safe procedure for patients with right-sided colon lesions.

## Introduction

A laparoscopy-assisted colectomy (LAC) is currently the standard management for benign or malignant colorectal lesions [1–4]. With advancements in surgical instruments and staple devices, total laparoscopic colectomy (TLC) is being performed increasingly for lesions arising from the left side of the colon and rectum. However, for lesions located on the right side, TLC is limited because of the need for intracorporeal hand-sewing of the anastomosis, which is technically more difficult than extracorporeal procedures. Moreover, increasing the surgical time and risk of intra-abdominal contamination with bowel content and tumor cells are major concerns. Many studies have demonstrated the advantages of laparoscopic right hemicolectomy with intracorporeal anastomosis (IA) in terms of short-term outcomes [5–8]. Some advantages of IA are that it helps avoid traction of the bowel through the small laparotomy wound and achieves greater lymph node yields and specimen lengths [8, 9]. We hypothesize that another advantage of IA is its potential assistance in the precise excision of a tumor, which contributes to a better surgical margin. However, currently, only few studies have analyzed the differences in specimen parameters with most studies presenting a longer specimen length acquired by using IA [8–11], and insufficient data are available regarding the resection length of tumor or the margin length according to different tumor locations, between IA and extracorporeal anastomosis (EA). Additionally, although comparable long-term oncologic outcomes between IA and EA have been reported [7, 12], the data are limited in disease-free status and cannot reflect the influence of local recurrence or distant metastasis distinctly.

In this study, the primary outcome was to analyze whether IA achieved a better specimen quality than EA by assessing the specimen parameters. The secondary outcome was to elucidate the postoperative morbidity, recovery, and short- and medium-term oncologic outcomes of laparoscopic right hemicolectomy with IA and compare the results to those of laparoscopic right hemicolectomy with EA.

## Materials and methods

### Patient selection

A total of 217 consecutive patients diagnosed with colon cancer who underwent an elective laparoscopic right hemicolectomy at Chang Gung Memorial Hospital between September 2016 and April 2018 were retrospectively analyzed. Among them, 2 patients without bowel anastomoses, owing to multiple comorbidities, were excluded.

### Operative technique

Pneumoperitoneum was created using the Veress needle technique or mini laparotomy at the umbilicus, which was decided according to the surgeon’s preference or the presence of a previous operative scar. Four ports were used including an 11-mm trocar below the umbilicus as a camera port, a 12-mm trocar in the left upper quadrant in the left mid-axillary line as a working port for the stapling device, and two 5-mm trocars in the left and right lower quadrants as working ports. A medial-to-lateral approach for mobilization of the right colon and corresponding mesentery was performed in all patients. A standard approach following the concept of complete mesocolic excision and central vascular ligation was performed. The ileocolic vessel was first ligated with the Hem-o-lok® System (Weck Closure Systems, Research Triangle Park, NC, USA) at its root from Superior mesentery vessels. Then, a medial-to-lateral, bottom-to-up dissection of retroperitoneum to lateral attachment of ascending colon and hepatic flexure was performed. After completely dividing the mesentery of the planned resected bowel, including the marginal artery, the ileocolic anastomosis was performed by either EA or IA according to the surgeon’s preference.

In the EA group, the umbilical wound was extended to a length of 5–7 cm, and a wound protector was used. The right side of the colon and terminal ileum were exteriorized through the midline incision, and the ileocolic anastomosis was created either using the antiperistaltic side-to-side stapler method or the isoperistaltic end-to-end hand-sewn method.

In the IA group, both ends of the transverse colon and terminal ileum were divided with Endo GIA staplers (Medtronic, Minneapolis, USA) laparoscopically. One of the following anastomosis methods was chosen according to the surgeon’s preference: (a) an isoperistaltic end-to-side hand-sewn method, (b) an isoperistaltic side-to-side staple method, and (c) an antiperistaltic side-to-side stapler method. For the isoperistaltic staple method, one enterotomy was created on the antimesenteric side of the ileum and colon, followed by insertion of the jaws of the staples and creation of the anastomosis. The common channel of the enterotomy was then closed with a barbed knotless suture by 3-0 V-Loc (Medtronic, Minneapolis, USA). For the antiperistaltic side-to-side stapler method, the enterotomy was created near the transection side, followed by a side-to-side staple anastomosis; the common channel of the enterotomy was also closed by stapling. After anastomosis, the specimen was extracted mainly through the Pfannenstiel incision or by extending the right lower quadrant port wound. For patients with a previous midline surgery, the umbilical wound was extended from the previous scar.

Natural orifice specimen extraction (NOSE) was also an alternative method. For female patients, a transvaginal or transrectal NOSE procedure was chosen according to the surgeon’s preference. For transvaginal approach, the posterior vagina was opened and a double-ringed wound protector (Alexis wound retractor; Applied Medical, Rancho Santa Margarita, CA, USA) was used to protect and shorten the vaginal canal after cleaning the vagina with povidone–iodine solution. After pulling out the specimen through the vaginal canal, the colpotomy incision was closed with an absorbable suture. For transrectal approach, rectosigmoid colon lumen was blocked using a bowel clamp followed by rectal irrigation with povidone–iodine solution. One enterotomy was made at the upper rectum, and a double-ringed wound protector was inserted to protect and shorten the rectum. After retrieval of specimen, the rectal opening was closed by barbed suture, and the air leak test was performed to confirm no mechanical failure. For male patients, transrectal NOSE can be used.

### Data collection

A retrospective analysis of data from the Colorectal Section Tumor Registry in Chang Gung Memorial Hospital, a prospectively designed database consisting of the records of postoperative patients who were consecutively and actively followed up, was conducted. Written informed consent was obtained from the patients prior to study participation. This study was approved by the Institutional Review Board of Chang Gung Memorial Hospital (approval number 202000106B0) and performed in accordance with the Declaration of Helsinki. All data were recorded in the hospital database and used for research purposes.

Preoperative variables, including age, sex, body mass index, comorbidity, history of previous abdominal surgery, American Society of Anesthesiologists (ASA) Class, and albumin level, were analyzed. The blood sample was obtained 1 week prior to the surgery. The operative parameters including the operative method, operative time, concomitant surgery, conversion to open surgery, blood loss, anastomosis methods, and specimen retraction site were collected. The pathological parameters including tumor location, tumor size, number of harvested lymph nodes, length of resection margins, T stage, and N stage were also analyzed. A subgroup analysis of the surgical margin according to the tumor location (cecum, ascending colon, hepatic flexure, and transverse colon) was also performed. The outcomes retrieved to compare the IA and EA groups included complications, mortality, length of hospital stay, readmission within 30 days of discharge, and pain scale scores at postoperative days 1 to 3. Time to first flatus or stool passage and time to tolerating a liquid and soft diet were also analyzed.

### Follow-up

All physicians at the author’s institution adopted similar follow-up routines and adjuvant treatment protocols. After primary tumor resection, all patients were subjected to a follow-up program that included outpatient visits every 3 to 6 months for physical examinations and CEA tests. Chest radiography, abdominal ultrasonography, or abdominal computed tomography (CT) imaging, in addition to colonoscopy, were performed after surgery and every 1 to 3 years whenever necessary. Follow-up status was confirmed postoperatively every 12 months by a team of three physicians and five specially trained nurses. Date of first recurrence was defined as the first date when the existence of local recurrence and/or distant metastases was confirmed by histology of biopsy specimens, additional surgery, and/or by radiological studies. The index date for survival calculation was the date of surgery for colon cancer. The definition of locoregional recurrence in our study is recurrent lesions within or adjacent to the original tumor location or dissection plane. Distant metastasis is defined as metastasis to non-locoregional sites like the liver, lung, or other organs. The presence of peritoneal recurrence was confirmed by either CT/positron emission tomography (PET) findings and/or surgical findings. The last follow-up date in this study was July 31, 2020.

### Statistical analysis

All parameters were analyzed using the Statistical Package for Social Sciences (SPSS) version 24 (IBM Corp., Armonk, New York, USA). Propensity score matching (PSM) was performed using a logistic regression model with the anastomosis method (IA vs. EA) set as the dependent variable. Patients were matched 1:1 by neighbor matching methods. The categorical variables were compared using Pearson’s chi-squared test, whereas continuous variables were compared using the independent sample *t* test. Survival analysis was performed using Kaplan-Meier curves with the log-rank test. *P* < 0.05 was considered statistically significant.

## Results

### Patient characteristics before and after matching

**Fig. 1:**
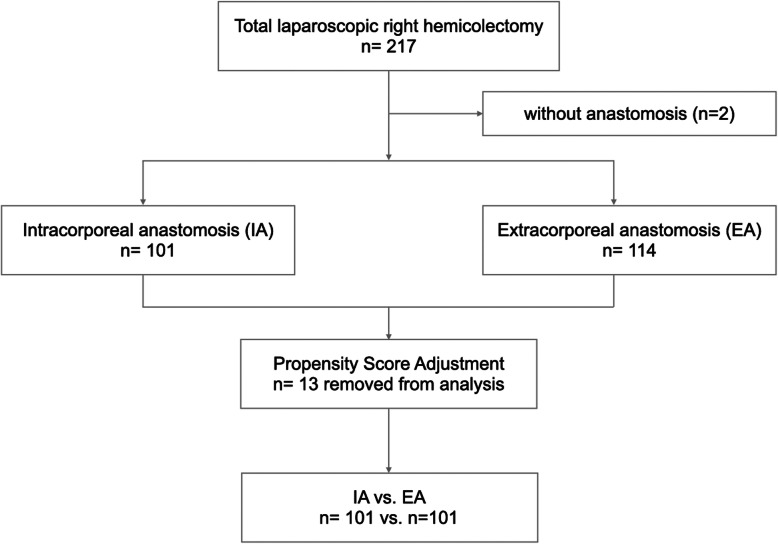
The flowchart of clinical data selection in this study

The total number of study patients and the number of patients in each study group before and after propensity score adjustment is presented in Fig. 1. Of the 215 patients included in the analysis, 114 and 101 patients underwent extracorporeal ileocolic anastomosis (EA group) and intracorporeal ileocolic anastomosis (IA group), respectively. The demographics of these patients are shown in Table 1. No significant differences were found between the EA and IA groups, except the preoperative albumin levels. There were more patients with low preoperative albumin levels in the EA group than in the IA group (< 3.5 g/dL, 13.3% vs. 3%, *p* = 0.007). After PSM, the study population included 101 patients in each group (Table 1). Baseline characteristics were not significantly different between the two groups.

**Table 1:** Demographic characteristics of patients undergoing intracorporeal and extracorporeal anastomosis before and after propensity score matching

	Before propensity score matching			After propensity score matching		
	IA^a^ (*n* = 101)	EA^b^ (*n* = 114)	*P* value	IA^a^ (*n* = 101)	EA^b^ (*n* = 101)	*P* value
Age (years)	64.43 ± 11.65	67.46 ± 13.84	0.086	64.43 ± 11.65	66.88 ± 12.79	0.155
Sex, *n* (%)						
Male	57 (56.4)	54 (47.4)	0.184	57 (56.4)	46 (45.5)	0.122
Female	44 (43.6)	60 (52.6)		44 (43.6)	55 (54.5)	
BMI^c^ (kg/m^2^)						
Mean	24.24 ± 3.66	24.14 ± 4.17	0.852	24.24 ± 3.66	24.45 ± 4.25	0.715
BMI^c^ > 30	6 (5.9)	8 (7.0)	0.749	6 (5.9)	8 (7.9)	0.580
Comorbidity, *n* (%)						
Hypertension	49 (48.5)	54 (47.4)	0.867	49 (48.5)	48 (47.5)	0.888
Cardiac disease	5 (5)	12 (10.6)	0.138	5 (5)	11 (11.0)	0.113
CVA^d^	1 (1)	2 (1.8)	1.000	1 (1)	2 (2.0)	0.621
Asthma	1 (1)	2 (1.8)	1.000	1 (1)	2 (2.0)	0.621
Diabetes	24 (23.8)	24 (21.2)	0.659	24 (23.8)	20 (20.0)	0.519
Cirrhosis	2 (2)	0 (0)	0.222	2 (2)	0 (0)	0.498
ASA^e^						
II	33 (32.7)	36(31.6)	0.864	33 (32.7)	35 (34.7)	0.766
III	69(67.3)	78(68.4)		68 (67.3)	66 (65.3)	
Previous abdominal operation	19 (18.8)	30 (26.3)	0.191	19 (18.8)	24 (23.8)	0.390
Albumin						
< 3.5 g/dl	3 (3)	15 (13.3)	0.007	3 (3)	3 (3)	1
≥ 3.5 g/dl	98 (97)	98 (86.7)		98 (97)	98 (97)	

^a^Intracorporeal anastomosis

^b^Extracorporeal anastomosis

^c^Body mass index

^d^Cerebrovascular accident

^e^American Society of Anesthesiologist

### Operative parameter

The operative parameters are listed in Table 2. There were no significant differences in the operative method, concomitant surgery, and blood loss between the two groups. Both groups had no case of conversion to laparotomy. The mean operative time was significantly shorter in the EA group than in the IA group (195.5 vs. 219.0 min, *p* = 0.034). There were various methods used to perform the ileocolic anastomosis in both groups, but the IA group predominantly used the method utilizing a staple with a barbed suture (56.4%). The incision made for specimen extraction was significantly different between the groups. In the EA group, all specimens were retracted from the trans-umbilical wound. In the IA group, a Pfannenstiel incision was made in 68.3% of the patients. The other incisions made included a trans-umbilical wound (8.9%), McBurney’s incision (18.8%), and NOSE (4%).

**Table 2:** Post-matching of operative parameters of patients undergoing intracorporeal and extracorporeal anastomosis

	IA^a^ (*n* = 101)	EA^b^ (*n* = 101)	*P* value
Operation			
RH^c^	96 (95)	91 (90.1)	0.180
Extended RH^c^	5 (5)	10 (9.9)	
Operative time, min	219.0 ± 77.1	195.5 ± 80.1	0.034
Concomitant surgery	7 (6.9)	13 (12.9)	0.158
Conversion to open	0 (0)	0 (0)	
Blood loss > 100 ml	6 (5.9)	10 (9.9)	0.297
Anastomosis methods			
Hand sewn	23 (22.8)	34 (33.7)	< 0.001
Total stapled	21 (20.8)	67 (66.3)	
Stapled and barbed suture	57 (56.4)		
Extraction site			
Trans-umbilical	9 (8.9)	101 (100)	< 0.001
Pfannenstiel	69 (68.3)		
NOSE^d^	4 (4)		
McBurney	19 (18.8)		

^a^Intracorporeal anastomosis

^b^Extracorporeal anastomosis

^c^Right hemicolectomy

^d^Natural orifice specimen extraction

### Pathological parameters

There were no significant differences in the tumor size, tumor location, number of harvested lymph nodes, stage of tumor invasion, and stage of lymph node invasion between the EA and IA groups (Table 3). The colon lengths were significantly different between the EA and IA groups (17.45 ± 5.81 and 20.36 ± 6.78 cm, respectively; *p* = 0.010); however, differences in the ileum length and nearest margin length were not statistically significant (Table 4).

**Table 3:** Post-matching of pathologic parameters of patients undergoing intracorporeal and extracorporeal anastomosis

	IA^a^ (*n* = 101)	EA^b^ (*n* = 101)	*P* value
Harvested lymph nodes	40.9 ± 19.3	41.1 ± 19.9	0.940
Tumor size (cm)			
Width	5.0 ± 9.7	4.3 ± 2.2	0.468
Length	4.5 ± 9.7	3.3 ± 1.5	0.242
Tumor location			
Cecum and appendix	19 (18.8)	16 (15.8)	0.369
Ascending colon	42 (41.6)	52 (51.5)	
Transverse colon and hepatic flexure	40 (39.6)	33 (32.7)	
T stage			
T0	5 (5)	2 (2)	0.778
T1	17 (16.8)	14 (13.9)	
T2	9 (8.9)	11 (10.9)	
T3	48 (47.5)	55 (54.5)	
T4a	17 (16.8)	15 (14.9)	
T4b	5 (5)	4 (4)	
N stage			
N0	57 (56.4)	72 (71.3)	0.286
N1a	15 (14.9)	9 (8.9)	
N1b	9 (8.9)	8 (7.9)	
N1c	2 (2)	1 (1)	
N2a	5 (5)	5 (5)	
N2b	13 (12.9)	6 (5.9)	

^a^Intracorporeal anastomosis

^b^Extracorporeal anastomosis

**Table 4:** Post-matching of the specimen length of patients undergoing intracorporeal and extracorporeal anastomosis according to tumor location

Tumor location	Specimen length (cm)	IA^a^ (*n* = 101)	EA^b^ (*n* = 101)	*P* value
All	Colon, mean	20.36 ± 6.78	17.45 ± 5.81	0.010
	Ileum, mean	5.70 ± 3.33	7.26 ± 7.90	0.760
	Nearest margin, mean	7.35 ± 3.67	6.62 ± 3.10	0.130
		IA^a^ (*n* = 19)	EA^b^ (*n* = 16)	
Cecum and appendix	Colon, mean	15.92 ± 4.63	14.33 ± 4.49	0.311
	Ileum, mean	8.70 ± 4.76	7.93 ± 6.11	0.686
	Nearest margin, mean	7.66 ± 4.87	6.82 ± 3.11	0.569
		IA^a^ (*n* = 42)	EA^b^ (*n* = 52)	
Ascending colon	Colon, mean	19.22 ± 6.92	16.96 ± 5.06	0.071
	Ileum, mean	5.61 ± 2.61	7.75 ± 9.73	0.175
	Nearest margin, mean	7.07 ± 2.98	7.33 ± 3.08	0.678
		IA^a^ (*n* = 40)	EA^b^ (*n* = 33)	
Transverse colon and hepatic flexure	Colon, mean	23.67 ± 5.95	19.75 ± 6.67	0.010
	Ileum, mean	4.49 ± 2.41	6.10 ± 4.88	0.075
	Nearest margin, mean	7.51 ± 3.81	5.40 ± 2.83	0.010

^a^Intracorporeal anastomosis

^b^Extracorporeal anastomosis

**Fig. 2:**
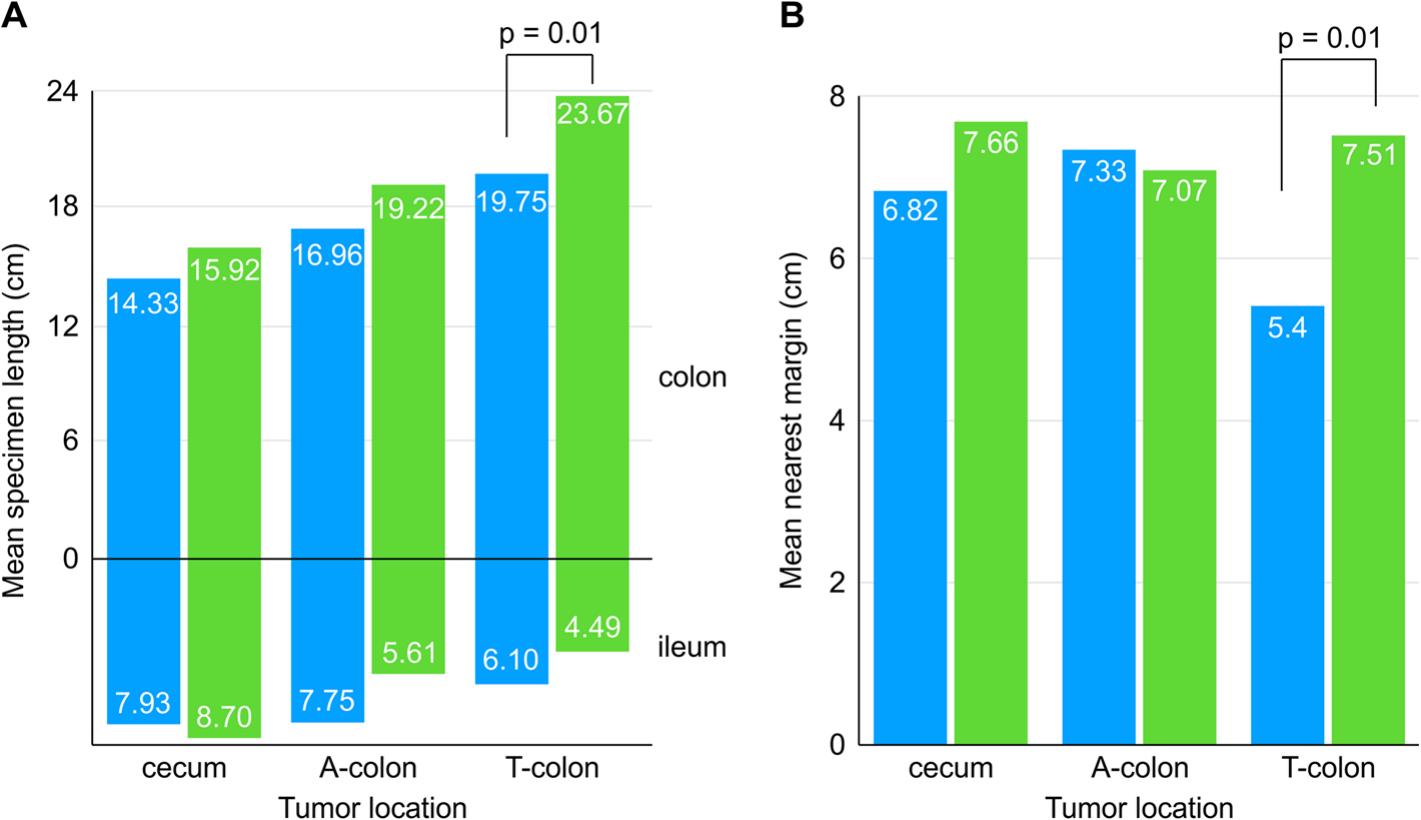
A comparison of the specimen length between extracorporeal anastomosis and intracorporeal anastomosis according to the tumor location. **a** Mean specimen length according to tumor location in patients that had extracorporeal anastomosis (EA group; blue bars) versus those that had intracorporeal anastomosis (IA group; green bars). **b** Mean nearest margin length according to tumor location, in the extracorporeal anastomosis group (EA group; blue bars) versus the intracorporeal anastomosis group (IA group; green bars)

In the subgroup analysis according to tumor location, a trend of increasing colon length but decreasing ileum length was observed in patients with tumors located from the cecum to the transverse colon in both groups (Fig. 2a). For tumors located from the hepatic flexure to the transverse colon, a significant difference in colon length was found between the EA and IA groups (19.75 ± 6.67 and 23.67 ± 5.95 cm, respectively; *p* = 0.010). There was also a significant difference in the length of the nearest margin between the EA and IA groups (5.40 ± 2.83 and 7.51 ± 3.81 cm, respectively; *p* = 0.010) (Fig. 2b). Although no significant difference was observed in the ileum length, we noted that the IA group had a shorter ileum distance from the transection line than the EA group (4.49 ± 2.41 vs. 6.10 ± 4.88 cm; *p* = 0.075).

### Short-term postoperative outcomes

The postoperative complications and short-term outcomes are listed in Table 5. The overall complication rates were similar between the IA and EA groups (8.9% vs. 10.9%, *p* = 0.818). No difference among severe complications (Clavien–Dindo Grade ≥ 3) was observed between the two groups (2% vs. 1%, *p* = 0.586). The major complication in both groups was prolonged postoperative ileus, with no significant difference observed between the groups. All patients recovered after conservative treatment. Three patients experienced intra-abdominal infection and received subsequent parenteral antibiotics treatment. Among them, one patient in the IA group presented with a pelvic abscess, which was confirmed by CT study and was readmitted within 1 month for percutaneous drainage. One patient in the IA group presented with dehiscence of anastomosis and required re-operation. Another patient in the EA group presented with dehiscence of anastomosis after discharge and required readmission for surgical management. One patient in the EA group had upper gastrointestinal bleeding and was readmitted for conservative treatment. One patient with a history of anxiety disorder visited emergency room for dyspnea and was admitted for conservative treatment. No mortality occurred in any of the groups.

**Table 5:** Post-matching of postoperative outcomes of patients undergoing intracorporeal and extracorporeal anastomosis

	IA^a^ (*n* = 101)	EA^b^ (*n* = 101)	*P* value
Total complications	10 (9.9)	11 (10.9)	0.818
Clavien–Dindo classification			
Grade 1	7	7	0.781
Grade 2	1	3	
Grade 3a	1	0	
Grade 3b	1	1	
Grade 4	0	0	
Grade 5	0	0	
Complication type			
Postoperative ileus	7 (6.9)	6 (5.9)	0.857
IAI^c^	1 (1)	2 (2)	
Dehiscence of anastomosis	1 (1)	1 (1)	
Wound infection	1 (1)	0 (0)	
Others	0 (0)	2 (2)	
Mortality	0 (0)	0 (0)	
Length of stay			
Mean	7.1 ± 3.9	8.7 ± 4.2	0.005
LOS^d^ ≤ 5 days	40 (39.6)	7 (6.9)	< 0.001
Readmission < 30 days	1 (1)	2 (2)	0.561
POD-3 data			
WBC^e^ ≥ 10000/mm^3^	47 (49)	28 (28.6)	0.004
CRP^f^ (mg/L)	88.1 ± 49.8	63.3 ± 41.7	< 0.001
Pain scale (visual analog scale)			
POD^g^ 1	4.2 ± 1.7	4.4 ± 1.9	0.430
POD^g^ 2	3.1 ± 1.3	3.4 ± 1.7	0.148
POD^g^ 3	2.4 ± 0.7	2.9 ± 1.4	< 0.001
Time to first flatus, days	2.2 ± 1.2	2.6 ± 1.1	0.027
Time to first bowel movement, days	3.5 ± 1.5	4.4 ± 2.0	< 0.001
Time to tolerate liquid diet, days	3.9 ± 2.6	4.9 ± 3.6	0.017
Time to tolerate soft diet, days	5.7 ± 3.2	6.7 ± 3.8	0.039

^a^Intracorporeal anastomosis

^b^Extracorporeal anastomosis

^c^Intra-abdominal infection

^d^Length of stay

^e^White blood cell

^f^C-Reactive protein

^g^Postoperative day

Patients who underwent IA had significantly lower pain scale scores at postoperative day 3 than those who underwent EA (2.4 vs. 2.9, *p* < 0.001). Better recovery was observed in the IA group than in the EA group in terms of time to first flatus (2.2 vs. 2.6 days, *p* = 0.027), time to first bowel movement (3.5 vs. 4.4 days, *p* < 0.001), time to tolerating a liquid diet (3.9 vs. 4.9 days, *p* = 0.017), and time to tolerating a soft diet (5.7 vs. 6.7 days, *p* = 0.039). The mean length of postoperative stay was significantly shorter in the IA group than in the EA group (7.1 vs. 8.7 days, *p* = 0.005). The proportion of patients in the IA group who had a length of stay (LOS) shorter than 5 days was 39.6%, whereas that in the EA group was 6.9% (*p* < 0.001).

The follow-up laboratory data on postoperative day 3 revealed that the IA group had more patients with leukocytosis than the EA group (white blood cell [WBC] ≥ 10000/mm^3^: 49% vs. 28.6%, *p* = 0.004) and a higher C-reactive protein (CRP) level (88.1 vs. 63.3 mg/L, *p* < 0.001).

### Long-term postoperative outcomes

**Fig. 3:**
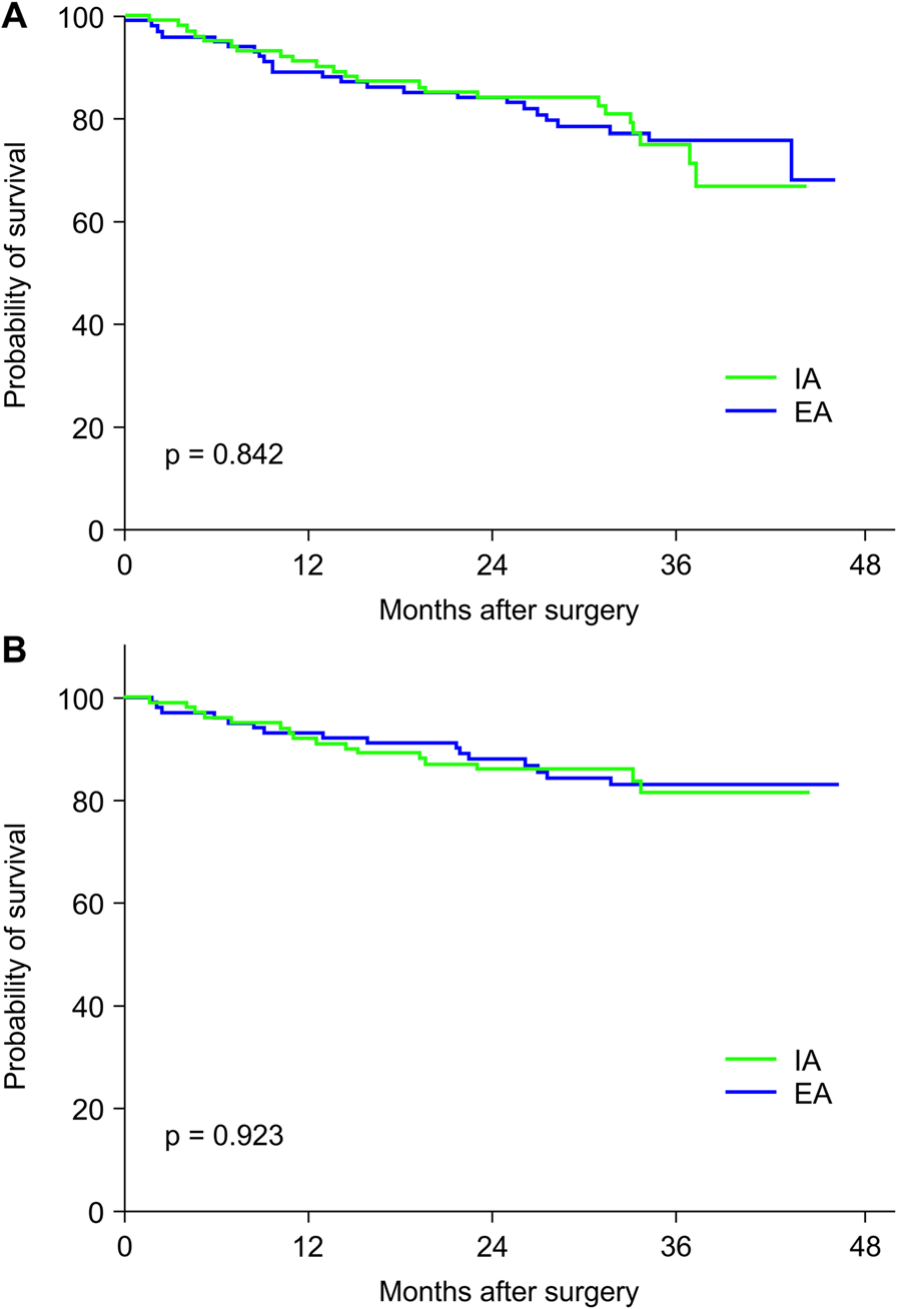
**a** Comparison of 3-year disease-free survival between patients who had extracorporeal anastomosis (EA group; blue bars) and those who had intracorporeal anastomosis (IA group; green bars). **b** Comparison of 3-year local recurrence-free survival between patients who had extracorporeal anastomosis (EA group; blue bars) and those who had intracorporeal anastomosis (IA group; green bars)

The long-term outcomes are listed in Table 6. The median follow-up times were 31.93 months and 36.7 months in the IA group and EA group, respectively. Four incisional hernias occurred during follow-up periods in the EA group, whereas no incisional hernia occurred in the IA group. The 3-year overall survival was not significantly different between the IA and EA groups (87.7% and 89.6%, respectively, *p* = 0.604). The 3-year disease-free survival was also not significantly different between the IA and EA groups (75.0% and 75.7%, respectively, *p* = 0.842). In subgroup analysis, there were 6 peritoneal recurrences found in the IA group and 8 in the EA group. The disease-free survival regarding peritoneal recurrence was not different at the 3-year follow-up between the IA and EA groups (81.6% and 82.9%, respectively, *p* = 0.923) (Fig. 3a and b).

**Table 6:** Post-matching of long-term outcomes of patients undergoing intracorporeal and extracorporeal anastomosis

	IA^a^ (*n* = 101)	EA^b^ (*n* = 101)	*P* value
Follow-up time, median months	31.93 (1.54–44.35)	36.70 (2.43–46.23)	0.001
Incisional hernia	0 (0)	4 (3.9)	0.121^C^
Peritoneal recurrence	6 (5.9)	8 (7.9)	0.580
Primary T3 lesion	2 (33.3%)	2 (25%)	1.000
Primary T4 lesion	4 (66.7%)	6 (75%)	
3-year overall survival	87.7%	89.6%	0.604
3-year disease-free survival	75.0%	75.7%	0.842
3-year peritoneal recurrence-free survival	81.6%	82.9%	0.923

^a^Intracorporeal anastomosis

^b^Extracorporeal anastomosis

^c^Fisher’s exact test

## Discussion

Nowadays, with innovations in surgical instrumentation, IA using linear staples and barbed suture material allows for the increasing application of total laparoscopic right hemicolectomies. In 2008, Bergamaschi et al. described one standardized IA procedure performed during a right colectomy for cancer, which showed favorable short-term results [5]. However, many surgeons still hesitate to use IA for laparoscopic right hemicolectomy, and EA remains the method of choice. Besides its technical difficulty, some concerns exist, including the possibility of increasing the risk of infection and exposure of tumor cell with this technique because the intestinal tract is opened during the anastomosis.

In this study, we compared the outcomes of patients who underwent laparoscopic right hemicolectomy for right-sided colon cancer using IA with that of those who underwent laparoscopic right hemicolectomy for right-sided colon cancer using EA. Surgical time was found to be significantly longer in the IA group than in the EA group. This is mainly attributed to the need for hand-sewing and knotting inside the abdominal cavity, and many surgeons required further training to perform intracorporeal sutures efficiently. The longer surgical time required to perform IA is also mentioned by several studies [5, 8, 13, 14]; however, we predict that this may shorten with increased surgical experience and the establishment of a standard approach. Given that EA requires grasping of the colon and ileum for subsequent anastomosis, a trans-umbilical incision is usually chosen and the length of the wound is inevitably longer; however, using IA, any incision site can be selected for specimen retraction and the length of the wound can be shorter for grasping one longer specimen. Many studies have revealed that patients with a lower abdominal incision experience less postoperative pain, a quicker return to ambulation and normal bowel function, and fewer pulmonary complications [15–18]. In our institution, we mainly choose the Pfannenstiel incision for tumor retraction because of its better postoperative outcomes and cosmetic results. In this study, the pain scale (visual analog scale) score showed a significant improvement on postoperative day 3 in the IA group. Moreover, some studies have shown a lower rate of incisional hernias with the use of the Pfannenstiel incision compared to that with an umbilical incision [16, 19, 20]. In our series, no incision hernia was found in the IA group at the 3-year follow-up, which is consistent with the findings of studies from western countries.

There are many advantages of IA for bowel management; one is the better visualization of both limbs of bowel. The EA method requires the bowel to be pulled through a small incision, which may cause small lacerations of the mesentery or torsion of the bowel while manipulating it during externalization. On the other hand, the IA method requires less mobilization of the bowel and can achieve better incisions that allow for a proper surgical margin to be obtained with better visualization using a direct-camera view. Less bowel manipulation is thought to enhance bowel function recovery. In this study, the times to first flatus, tolerating a soft diet, and bowel movement were significantly shorter in the IA group, which is similar to that found in other studies [8, 12, 21]. Another advantage of IA is that it allows for better alignment of the bowel during anastomosis with isoperistaltic ileocolic anastomosis and less inversion of the ileum and its mesentery may also contribute to better recovery of bowel function.

During the pathological review, the number of harvested lymph nodes was not significantly different between the IA and EA groups in our study, although some studies demonstrated a higher number of retrieved lymph nodes with the IA method [8, 12]. Only a few studies have compared the difference in specimen length and surgical margin between the IA and EA groups. Magistro et al. compared these parameters in 40 patients undergoing a TLC with that of 40 patients undergoing a LAC and found a longer specimen length in the IA group (37.7 ± 12 cm vs. 29.8 ± 8.2 cm, *p* = 0.001) [8]. Another study by Biondi et al. revealed that better specimen and vascular pedicle lengths are achieved using the IA method. In this study, a greater colon length was achieved with the IA method than with the EA method (20.36 vs. 17.45 cm, *p* = 0.01) [9]. In a single-blind, randomized clinical trial conducted by Bollo et al., the colon length was also longer in the IA group than in the EA group (25.3 vs. 22.7 cm, *p* = 0.026) [10]. In the subgroup analysis, we noted no significant difference in colon length when the lesions were in the cecum or appendix. Interestingly, the further the lesion was from the cecum, the greater the difference in colon length was between the IA and EA groups. When the tumor was located at the hepatic flexure or transverse colon, the colon length and nearest margin were greater when using the IA method. This finding may be explained by the greater visualization of the bowel, achieving a more precise incision and minimizing bowel compromise during resection by avoiding the need to exteriorize a thick specimen through a small laparotomy incision with IA.

To date, there is only one study that compared the postoperative laboratory data of the IA and EA groups. Mari et al. compared preoperative and serial postoperative serum inflammatory markers between 30 patients who underwent a TLC and 30 patients who underwent a LAC and found significantly lower interleukin-6, CRP, and WBC levels on postoperative days 1, 3, and 5 in the IA group [21], indicating that the IA group had a lower surgical stress response that might play a role in bowel recovery. In contrast, this study revealed significantly higher WBC and CRP levels on postoperative day 3 in the IA group. Although these high inflammatory markers were present, there was no difference in the incidence of postoperative complications, such as intra-abdominal infection, abscess formation, wound infection, or anastomosis insufficiency, between the groups. The incidence of postoperative ileus was also comparable between the two groups, suggesting that the risk of infection and postoperative complications was not increased when opening the intestinal tract during IA.

In this study, the mean LOS after surgery was significantly shorter in the IA group, especially a LOS less than 5 days. The shorter LOS represents an overall better recovery after surgery with the IA method, which includes less wound pain, less stress on the bowel, and better recovery of bowel function. Many systemic reviews and meta-analyses have demonstrated similar findings [22–27]. The IA method has the following advantages: lower analgesic use, faster time to bowel movement and flatus passage, shorter time to solid food intake, and shorter length of hospitalization. The postoperative complications were comparable between the two methods [7, 8, 10, 24–27].

Hanna et al. demonstrated comparable 5-year overall survival (66% vs. 78%, *p* = 0.698) and disease-free survival (86% vs. 78%, *p* = 0.999) between the IA and EA groups, comprising of 195 patients undergoing a laparoscopic right hemicolectomy [12]. Another study by Lee et al. also showed comparable overall survival (71% vs. 76%, *p* > 0.05) and disease-free survival (82% vs. 85%, *p* > 0.05) between the IA and EA groups at the 3-year follow-up [7]. In the present study, no significant difference in overall survival and disease-free survival was observed between the IA group and EA group. It is of concern that tumor cell dissemination while opening the intestinal tract in an environment of high-pressure pneumoperitoneum may cause an increased rate of peritoneal recurrence. To survey whether peritoneal recurrence is increasing because of opening the intestinal tract during the IA procedure, we focused on the occurrence of peritoneal seeding during the follow-up. Among all participant studies, there were equivalent numbers of peritoneal seeding detectable by CT/PET survey and/or re-operation between the IA and EA groups, indicating no increased risk of tumor cell dissemination even if we open the intestinal tract inside the peritoneal cavity. Ambe et al. analyzed the cytology specimens acquired from the endoscopic retrieval bag after intracorporeal resection of the colorectal cancer laparoscopically and found no malignant cells. Furthermore, peritoneal recurrence was not observed during the 14-month follow-up [28]. We noted that the primary T stage of the resected tumor in our series was T3 or T4, which may imply that the tumor factor itself is more critical than the surgical factor.

The strength of this study is that, to our knowledge, we are the first to compare, in detail, the length of specimens and margins according to the tumor location between patients who underwent IA and those who underwent EA to confirm the hypothesis that IA offers a precise resection of the specimen. Moreover, we are the first to compare whether peritoneal recurrence is increased with the IA procedure to answer the question whether surgeons should avoid opening the intestinal tract during the surgery for better oncological results. This study is limited by its retrospective nature; however, by applying PSM, the demographic characteristics of both groups used in the analysis were the same. Another limitation in this study is the small sample size and the oncological results may not be as reliable as larger trials. Long-term results by larger prospective trials are desirable to confirm these findings.

## Conclusions

In conclusion, laparoscopic right hemicolectomy with IA offers a variety of wound incision sites for tumor retraction, achieves better postoperative recovery without increasing the incidence of complications, and allows for a more precise incision that achieves a better surgical margin. The overall survival, disease-free survival, and the rate of peritoneal recurrence were comparable between IA and EA procedures. Thus, IA can be considered a safe procedure for patients with right-sided colon lesions.

## Data Availability

The datasets generated and analyzed during the current study available from the corresponding author on reasonable request.

## Abbreviations

LAC: Laparoscopy-assisted colectomy
TLC: Total laparoscopic colectomy
IA: Intracorporeal anastomosis
EA: Extracorporeal anastomosis
ASA: American Society of Anesthesiologists
PSM: Propensity score matching
NOSE: Natural orifice specimen retraction
LOS: Length of stay
CEA: Carcinoembryonic Antigen
PET: Positron emission tomography
